# Investigation of protective level of rabies antibodies in vaccinated dogs in Chennai, India

**DOI:** 10.1002/vro2.8

**Published:** 2021-04-05

**Authors:** Gowri Yale, Sampada Sudarshan, Shaheen Taj, Ganesan Irulappan Patchimuthu, Bharathi Vijaya Mangalanathan, Ashwin Yajaman Belludi, Madhusudana Narayan Shampur, Tirumurugaan Gopalan Krishnaswamy, Stella Mazeri

**Affiliations:** ^1^ Mission Rabies Veterinary Hospital Complex Panaji Goa India; ^2^ Department of Neurovirology National Institute of Mental Health and Neurosciences Bangalore Karnataka India; ^3^ Department of Veterinary Medicine Apolllo College of Veterinary Medicine Jaipur Rajasthan India; ^4^ Department of Veterinary Preventive Medicine Madras Veterinary College Tamil Nadu Veterinary and Animal Sciences University Chennai Tamil Nadu India; ^5^ Department of Neurovirology National Institute of Mental Health and Neurosciences Bangalore Karnataka India; ^6^ Zoonoses Research Laboratory Centre for Animal Health Studies Tamil Nadu Veterinary and Animal Sciences University Chennai Tamil Nadu India; ^7^ Division of Genetics and Genomics The Roslin Institute and The Royal (Dick) School of Veterinary Studies The University of Edinburgh, Midlothian UK

## Abstract

**Background:**

Rabies is still endemic in India causing an estimated 20,000 human deaths a year. Free roaming dogs and unvaccinated owned dogs play a major role in the maintenance of the disease. Dog vaccination is the most crucial aspect of rabies prevention and control strategies; therefore vaccine immunogenicity and longevity are important determinants of the efficiency of rabies control efforts.

**Methods:**

In this study at Madras Veterinary College, India, a total of 297 serum samples were collected from owned dogs that were vaccinated against rabies. Data regarding age, gender, breed, neuter status and last date of vaccination were collected at the time of blood collection. The level of rabies virus neutralising antibodies in the sera of these dogs was measured through rapid focus fluorescence inhibition test. The factors associated with protective level of rabies antibodies in vaccinated dogs were investigated through multivariable regression analysis.

**Results:**

This cross‐sectional investigation shows that only 40% (119/297) of the all the dogs in the study showed presence of protective level of anti‐rabies antibodies, and 40% (72/180) of the dogs vaccinated within the last year showed presence of protective levels of antibodies causing concern about rabies vaccine quality and its impact on rabies control. The study also shows that older and neutered dogs are more likely to have protective titre among vaccinated dogs, while non‐descript breed dogs are less likely to have a protective titre compared to pure breeds.

**Conclusion:**

In this study 60% (108/180) of young prima dogs and adult dogs did not show protective levels of antibodies within the year of last rabies vaccination, although they had previous vaccination history. This high percentage of apparent non‐responders is a cause of concern of administration, distribution, storage, potency and quality management of vaccines in India.

## INTRODUCTION

Dogs are the reservoir of rabies in most rabies endemic countries around the world.^1^ India is a rabies endemic country with the largest estimated annual rabies deaths. It is estimated to account for 35% of the world's rabies deaths approximating to 20,000 deaths a year, causing an annual loss of 2.3 billion USD through premature death, bite treatment, loss of labour, livestock losses and post‐exposure prophylaxis.^2^ Dogs continue to be the primary source of rabies in India and make up 96.2% of the animal bites reported of which 75% are reported to be from free roaming dogs.^3^

Dog populations in India can be broadly categorised into three groups: 1) owned or pet dogs 2) community or partially owned and 3) free roaming. Owned or pet dogs are generally confined with restricted movement and are always supervised outside home. These dogs are completely dependent on their owner for food and shelter. They receive regular veterinary care and receive rabies vaccination when the owner takes their dogs to a private or government veterinary establishment or if there is a door to door mass dog vaccination campaign. Community or partially owned dogs are dogs that are partially restricted or not supervised. They are partially dependent on people for food and shelter. These dogs do not receive any veterinary care. Free roaming dogs are completely free and do not depend on people for food or shelter and do not receive any veterinary care. The second and third groups of dogs have a chance of receiving a shot of rabies vaccine only if there is mass dog vaccination or mass dog sterilization effort in the region.

Free roaming and community dogs are seen as a public menace for various reasons besides biting and rabies.^4^ There is also a concern of animal welfare when people respond to the menace in inhumane ways; therefore the public sector began focused efforts towards rabies and dog population control through humane methods. These efforts are apparent through mass dog sterilization initiatives or catch‐neuter‐vaccinate‐release programs in urban and semiurban areas in India,^5^, ^6^ which started in 1992.^7^ Mass dog vaccination campaigns targeting control of canine rabies are also gaining gradual momentum in India.^8^ Rabies elimination through mass dog vaccination has been demonstrated multiple times before,^9^, ^10^ and World Organisation for Animal Health (OIE) and World Health Organisation (WHO) recommend canine rabies control as a strategy in eliminating dog mediated human rabies.^11^, ^12^ The National Rabies Control Programme under Government of India is also working in tandem with the global goal ‘zero by 30′ initiated by the tripartite to achieve zero human rabies deaths by the year 2030.^13^ The quality of the vaccine used to control rabies in any group of dogs (owned, free roaming, community) and in any delivery strategy (veterinary clinic, door to door, mass dog vaccination) remains to be one of the most critical components of this global goal.^14^

Since the quality of canine rabies vaccines is one of the most critical factors in the efforts of rabies control, OIE and WHO recommend that canine vaccines should confer protective immunity for at least 1 year^11^ and 2 years,^12^ respectively. This study explores the presence and factors associated with protective level of rabies antibody titres (PLORAT) in a vaccinated dog population sample in Chennai, India.

## MATERIAL AND METHODS

### Ethics statement

Clearance and approval of study protocol were obtained from the Institutional Animal Ethics Committee of Tamil Nadu Veterinary and Animal Sciences University for this study (1252/B/DFBS/IBSC/2011/ dated 31/10/2011) which adheres to the Committee for the Purpose of Control and Supervision of Experiments on Animals, Government of India. None of the authors have any competing interest in this study.

### Sample collection

Blood was collected from dogs presented to Madras Veterinary College outpatient section of the Clinical Medicine Department, a government veterinary teaching hospital in Chennai City with a primary to tertiary case load. Sera samples were collected from 8 September 2011 to 21 September 2012. All the samples in this study were collected from dogs that had an owner who cared for and took full responsibility for its health. Only eligible dogs of owners who understood the objective of the study and consented were included in the study. Dogs were eligible for inclusion in the study if they were apparently healthy with good body score condition and had been vaccinated for rabies any time before 3 weeks to the day of blood collection. Vaccination cards were used as a proof of vaccination whenever it was possible otherwise most records for the date of vaccination are based on the memory of the dog owners. If the owner could not remember the last date of vaccination, the dog was not included in the study. Additionally the dogs included needed to have their prima rabies vaccine above the age of 3 months as recommended by vaccine labels. All dogs included in the study were vaccinated by vaccines approved by the Veterinary Cell, Central Drugs Standard Control organisation, Directorate General of Health Services (DGHS), Ministry of Health and Family Welfare, Government of India. Dogs were excluded if they had never received rabies immunization and/or were clinically unhealthy. All dogs except 13 puppies aged less than 6 months had received at least two doses of rabies vaccine at the time of blood collection. The brand names of the vaccines were not recorded. Data regarding details of the dogs’ age, gender, breed, neuter status and last date of vaccination were collected at the time of blood collection using a structured questionnaire by the author. The breed of the dogs was assigned visually by the author from veterinary expertise.

### Laboratory analysis

Rapid fluorescence focus inhibition test (RFFIT) was conducted to estimate rabies antibody level in the sera of the dogs. This was performed as per the WHO advocated procedure^15^ with some modifications at the Department of Neurovirology, National Institute of Mental Health and Neurosciences (NIMHANS), Bangalore, a WHO Collaborating Centre for Reference and Research on Rabies. Instead of tissue culture chambers, 96‐well flat‐bottomed tissue culture plates were used, and the cell line used was Baby Hamster Kidney (BHK −21). The virus used was a CVS strain adapted to grow in BHK‐21 cells, and the dose used was 100 FFD_50_. The highest dilution of serum showing 50% inhibition of fluorescent foci in the infected cells was taken as the titre of the serum, which was converted to international units (IU/ml) by comparison to an in‐house reference sera calibrated against the 2nd international reference serum with a unitage of 30 IU/ml (obtained from the National Institute of Biologicals, UK). Rabies neutralising antibody titre of 0.5 IU/ml is defined ‘protective’ by WHO and OIE.^11^

### Statistical analysis

Data were analysed using the statistical software R 3.4.2.^16^ Two multivariable regression models were built in order to identify factors associated with rabies antibody titres. A multivariable logistic regression model was used to identify factors associated with a dog having a protective antibody titre (>0.47IU/ml), while a multivariable linear regression model was used to identify factors associated with antibody titre levels. To deal with missing values in the dataset, any variables with missing values had an extra category added called 'missing' to avoid rows being automatically removed by regression analysis. This enabled the use of the whole dataset, therefore avoiding bias due to absence of data.

Vaccinated dogs were grouped into three categories depending on duration of time that had lapsed from the date of last vaccination to time of blood collection: 1) vaccinated within a year 2) vaccinated between 1 and 3 years and 3) more than 3 years ago. Dogs were also divided into three groups based on their body weight and breed: 1) large breed included dogs with defined breed and weighed more than 25 kg, 2) small breed included dogs with defined breed and weighed less than 25 kg and 3) non‐descript (ND) included mixed breed dogs, dogs that were not defined as a breed, and the free roaming dog variety popularly called “Indy”.

Univariable analysis was used as a pre‐screening for selecting variables that would be considered in the multivariable linear and logistic regression models, respectively. Variables with a *p*‐value < 0.15 were considered in the multivariable models. Variable selection was carried out using the dredge function from the MuMIn R package,^17^ whereby models with all variable combinations are fit. The final models were selected based on corrected Akaike information criterion (AICc). Models with an AICc within 2 units of the lowest AICc were averaged to give the final model.

## RESULTS

**FIGURE 1:**
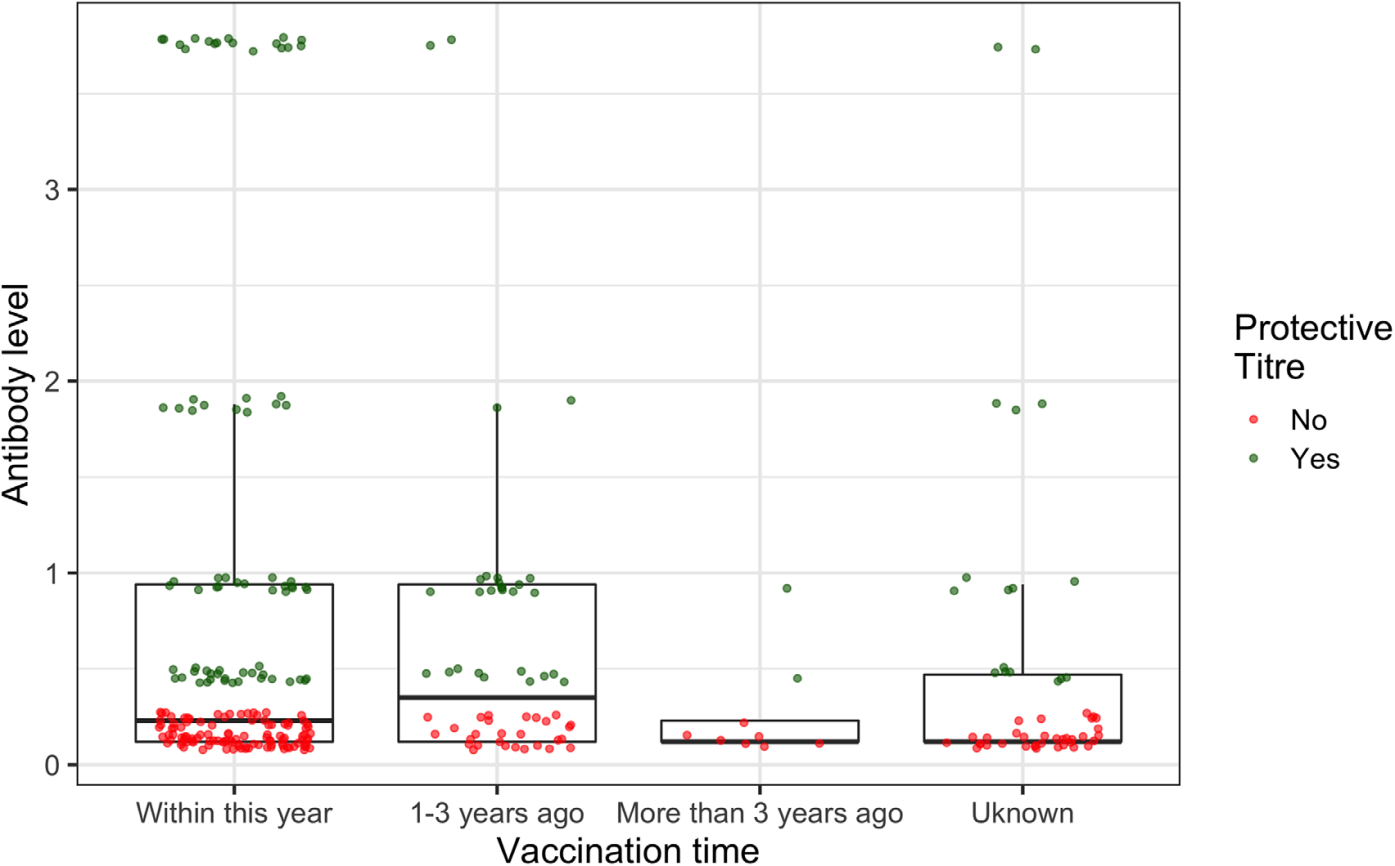
Distribution of dogs with protective level of rabies antibody titres (PLORAT) sorted according to the date of last vaccination

A total of 297 blood samples collected from dogs satisfied the selection criteria, of which, 119 (40%) vaccinated dogs showed PLORAT, while 60% did not. Table 1 and Figure 1 show the proportion and distribution of dogs with PLORAT according to the date of last vaccination. Only 40% (72/180) of dogs vaccinated within the last year showed PLORAT, 50% (28/56) of the dogs vaccinated during the last 1–3 years showed PLORAT, 22.2% (2/9) of dogs vaccinated more than 3 years ago showed PLORAT and 32.7% (17/52) of dogs with unknown date of vaccination showed PLORAT. The proportion of the dogs that showed a PLORAT and were vaccinated anytime within 3 years was 42.4% (100/236).

**TABLE 1:** Proportion of dogs with protective level of rabies antibody titres (PLORAT) sorted according to the date of last vaccination

Vaccination time	Protective titre	Total	Percentage	95% CI
Within this year	72	180	40.0	32.78–47.55
1–3 years ago	28	56	50.0	36.34–63.66
More than 3 years ago	2	9	22.2	2.81–60.01
Unknown	17	52	32.7	20.33–47.11

Univariable analysis results for both logistic and linear regression are shown in Tables 2 and 3, respectively. These show all variables considered for analysis. Variables which satisfied the *p* < 0.15 criterion were considered for inclusion in the multivariable models.

**TABLE 2:** Univariable analysis for logistic regression model predicting presence of protective antibody titre

Variable	Odds ratio	95% confidence interval	*p*‐value
Age (months)	1.01	1–1.02	0.001
Sex			
Female	1	Reference category	
Male	0.92	0.58–1.46	0.72
Breed			
Non‐descript	1	Reference category	
Breed: Small	2.35	1.06–5.24	0.036
Breed: Large	1.76	0.91–3.43	0.094
Neuter status			
Entire	1	Reference category	
Neutered	4.88	1.53–15.51	0.007
Number of days since last vaccination	1	1	0.919
Time since last vaccination			
Within this year	1	Reference category	
1–3 years ago	1.5	0.82–2.74	0.187
More than 3 years ago	0.43	0.09–2.12	0.299
Vaccination frequency			
Irregular	1	Reference category	
Regular (annually)	0.95	0.5–1.8	0.864

**TABLE 3:** Univariable analysis for linear regression model predicting antibody titre

Variable	Estimate	95% confidence interval	*p*‐value
Age (months)	0	0‐0.01	0.03
Sex			
Female	1	Reference category	
Male	−0.05	−0.27–0.17	0.64
Breed			
Non‐descript	1	Reference category	
Breed: Small	0.53	0.17–0.89	0.004
Breed: Large	0.19	−0.1–0.48	0.204
Neuter status			
Entire	1	Reference category	
Neutered	0.86	0.39–1.34	0
Number of days since last vaccination	0	0‐0	0.305
Time since last vaccination			
Within this year	1	Reference category	
1–3 years ago	−0.12	−0.42–0.17	0.416
More than 3 years ago	−0.46	−1.13–0.2	0.173
Vaccination frequency			
Irregular	1	Reference category	
Regular (annually)	0.07	‐0.26–0.4	0.676

**FIGURE 2:**
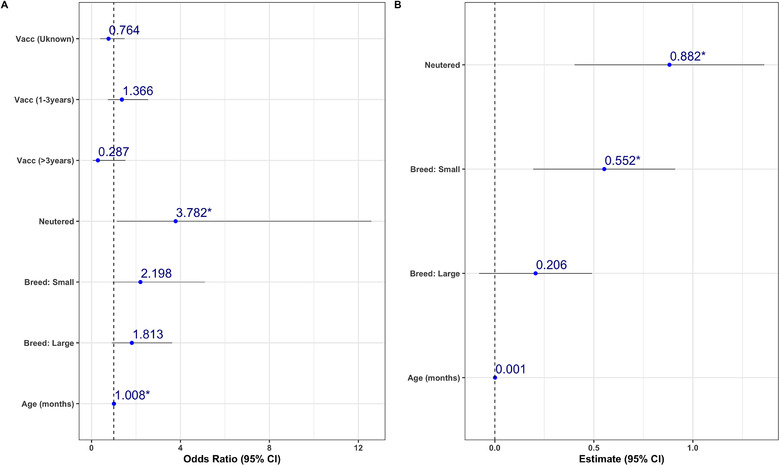
Results of the multivariable logistic (a) and linear (b) regression models predicting presence of protective antibody titre and antibody titre levels, respectively. Odds ratios (dots) and 95% confidence intervals (lines) are shown for each model

Multivariable logistic regression (Figure 2a) showed that neutered dogs have almost four times greater odds of protective antibody titres over intact dogs. Compared to dogs vaccinated during the last year, dogs vaccinated more than 3 years ago have almost four times lower odds of having a protective titre. Also, compared to ND breed dogs, both small and large breed dogs have around twice the odds of having a protective titre. The odds of having a protective titre also increased with age. In other words, older dogs have greater odds of having protective titres.

Multivariable linear regression (Figure 2b) supported these results by showing that neutered dogs had higher titres than entire dogs. Similarly, antibody titre increased with age. Lastly, ND breed dogs had lower titres than small and large breed dogs. Numerical results of the regression models can be found in Tables 4 and 5.

**TABLE 4:** Multivariable logistic regression model predicting presence of protective antibody titre

Variable	Odds ratio	95% confidence interval	Standard error	*p*‐value
Age (months)	1.008	1.002–1.014	0.003	0.009
Neuter status: Entire	1	Reference category		
Neuter status: Neutered	3.782	1.136–12.587	0.611	0.030
Breed: Non‐descript	1	Reference category		
Breed: Small	2.198	0.947–5.101	0.428	0.067
Breed: Large	1.813	0.906–3.628	0.352	0.093
Time since last vaccination: Within the year	1	Reference category		
Time since last vaccination: 1–3 years ago	1.366	0.731–2.550	0.317	0.328
Time since last vaccination: More than 3 years ago	0.287	0.054–1.524	0.848	0.143
Time since last vaccination: Missing	0.764	0.390–1.494	0.341	0.431

**TABLE 5:** Multivariable linear regression model predicting antibody titre levels

Variable	Estimate	95% confidence interval	Standard error	*p*‐value
Breed: Non‐descript	1	Reference category		
Breed: Small	0.552	0.194–0.911	0.182	0.003
Breed: Large	0.206	−0.080–0.491	0.145	0.158
Neuter status: Entire	1	Reference category		
Neuter status: Neutered	0.882	0.402–1.361	0.244	<0.01
Age (months)	0.001	−0.0014444–0.004	0.001	0.367

## DISCUSSION

In our study 60% of young prima dogs and adult dogs did not show protective levels of antibodies within the year of their last rabies vaccination, although they had a previous vaccination history. This high percentage of apparent non‐responders is a cause of concern. There are various reasons why vaccination of dogs with inactivated rabies vaccines does not lead to neutralising antibody levels considered to be protective including intrinsic animal and extrinsic vaccine factors.^18^ The factors that can be completely controlled are the extrinsic factors that pertain to vaccine quality by manufacturers, cold chain maintenance by distributor and final stage suppliers, storage and administration by veterinarians.

Inadequate levels of protective antibody in the first year of vaccination after a single dose of primary rabies immunization have been reported in the range of 3.1%–51%.^19^, ^20^, ^21^, ^22^, ^23^ Studies have therefore emphasised the need of a booster dose of rabies vaccine in puppies and dogs under the age of one to reach protective levels of antibodies within the first year.^23^, ^24^

OIE and DGHS of India recommend that canine rabies vaccines should confer a minimum of 1 year immunity in dogs, and yet only 40% of the dogs vaccinated within a year showed protective antibody titres depicting a 60% failure to reach PLORAT. Most vaccine labels promise 3 years of immunity, and yet only 42.4% (100/236) of the dogs that were vaccinated within a total of 3 years showed protective levels of immunity.

Absence of protective levels of rabies virus neutralising antibody titres in vaccinated dogs does not necessarily indicate that they are susceptible to rabies infection if challenged but there is inadequate information regarding the outcome in dogs that merely seroconvert and do not reach protective levels. Kennedy et al^25^ discussed that the dog's total immunity does not reduce but only shifts from a more dominant IgM to a more IgG‐based immunity. Additionally, the role of cellular immunity and antibodies other than neutralising antibodies that contribute towards immunity from rabies requires further investigation to understand the protection that is observed sometimes in animals that do not show PLORAT or animals that show neutralising antibodies without previous vaccination.^26^, ^27^, ^28^ Our study reports results from RFFIT, which is a gold standard technique for virus neutralising antibody technique.^29^

The humoral response to parenteral anti‐rabies vaccination shows a classic profile where the immune response begins with a latent phase followed by an exponential phase and a plateau, then a decrease in the antibody levels. The peak is generally reached between 4 and 6 weeks and remains stable throughout the year with a short period of low phase between 16 and 25 weeks before waning down gradually over the following year.^19^, ^23^ Since the exact date of vaccination is unknown in most of the cases in this study, samples could have been collected in the low phase of immune response leading to high proportion of low responders in the first group; dogs were vaccinated within a year.

As age increased, odds of a PLORAT increased. This can be explained by the well‐established fact that adult dogs and dogs that have received multiple doses of vaccination over the years have a better chance to result in PLORAT.^19^, ^30^ Additional detailed information on previous vaccinations of the dogs in this study would contribute significantly to the results obtained; the authors recognise this as a limitation in the study since this information was not precisely available from the dog owners.

Dogs that were vaccinated 1–3 years ago showed contradicting results where 50% of them tested with PLORAT leading the category to slightly higher odds of PLORAT compared to dogs vaccinated within the last year. These results are suspected to be skewed by the possibility that the dogs presented with last dose of vaccination 1–3 years ago to be older dogs who had received multiple doses of annual rabies vaccination previously.

Neutered dogs in the study showed greater odds of PLORAT than intact dogs, a result that was comparable with work by Kennedy et al^25^ which indicated that neutering increased the chances of PLORAT by 22%. Nevertheless, there is still debate about the effect of neutering on health, and the results could be confounded by the fact that neutered dogs were probably cared for better than dogs that were not; assuming neutering reflects responsible ownership and better care.^30^, ^31^

Our study shows a very high percentage of low responders (less than PLORAT) in spite of all dogs included in the study being owned. Owned dogs have regular access to food, veterinary care and shelter compared to free roaming dogs that do not have a healthy diet, veterinary care or comfortable shelter. Additionally our analysis shows that the ND dogs in our group had lower odds of achieving PLORAT compared to pure‐bred dogs, although mixed breeds have been known to show better immune response to vaccine in other reports.^23^ A possible explanation might be that owners of ND dogs resorted to lower priced, lower quality vaccines.^32^

A mass dog vaccination campaign in India costs approximately 250 INR (3.5 USD) per dog with 70% of the total being operational costs (staff salaries, vehicles, fuel, equipment, needle‐syringe etc). The cost of the vaccine is only approximately 30% of the campaign expenditure,^33^ but a substandard vaccine has the potential to jeopardise the entire campaign as previously experienced in other mass dog vaccination efforts.^34^ Additionally, dogs in mass vaccination programs receive a single dose of vaccine with no chance of a booster within the year. Hence the quality of vaccines used in these programs should be high enough to induce significant protection with a single immunization.

Mass dog vaccination campaigns and veterinary public sector must have the power of judgement on the quality of vaccine during procurement, factoring in duration of immunity, thermos‐stability and efficacy^35^, ^36^ and not be completely bound by the tender process of lowest bidder as it may jeopardise the quality of the vaccine purchased and hence the whole control effort. A more stringent vaccine quality control measure is required to be enforced for efficient infectious disease control including random serological potency assays^36^, ^37^ by government appointed independent agencies at end user stage to monitor quality of the vaccines administered. Higher proportion of protective levels of antibody titres has been reported in other studies demonstrating the possibility of better seroconversion and maintenance of adequate levels of antibodies.^28^, ^38^, ^39^ A randomised control trial to compare potency of different vaccine brands available in India would challenge this discussion providing insight towards better solutions.

## CONCLUSION

In this study 60% of vaccinated dogs showed inadequate protective levels of titres within the year of last rabies vaccination in 180 vaccinated dogs in Chennai, India. This proportion is far higher than other reports from similar studies.^40^, ^41^, ^42^ This is not only a concern for rabies control efforts in the canine population but also increases the risk of rabies in humans who may not seek post‐exposure prophylaxis after a bite from a vaccinated dog. It is crucial that vaccines selected for use by the veterinary public sector and for mass dog vaccination campaigns are of high quality and retain potency until administration. Regular investigation through neutral agencies is required to ensure quality of vaccines is maintained from production to administration.
